# A Comorbid Rat Model of Neuroendocrine-Immune System Alterations Under the Impact of Risk Factors for Stroke

**DOI:** 10.3389/fnagi.2021.827503

**Published:** 2022-01-20

**Authors:** Bailiu Ya, Xuezhi Li, Jingyi Wang, Mingsheng Zhao, Ting Yu, Haiying Wang, Qing Xin, Qinqin Wang, Xin Mu, Xuanyu Dong, Yang Gao, Huabao Xiong, Hui Zhang

**Affiliations:** ^1^Department of Physiology, Basic Medical School of Jining Medical University, Jining, China; ^2^Shandong Key Laboratory of Behavioral Medicine, Shandong Collaborative Innovation Center for Diagnosis, Treatment and Behavioral Interventions of Mental Disorders, School of Mental Health, Jining Medical University, Jining, China; ^3^Institute of Immunology and Molecular Medicine, Jining Medical University, Jining, China; ^4^Department of Histology and Embryology, Basic Medical School of Jining Medical University, Jining, China

**Keywords:** carotid atherosclerosis, hypercholesterolemia, neuroendocrine-immune, inflammation, complex animal model, comorbidity

## Abstract

Hypercholesterolemia and carotid atherosclerosis contribute to the etiology of stroke. However, there has been a lack of appropriate comorbid animal models incorporating some of the ubiquitous characteristics that precede strokes. Curcumin is a natural active polyphenolic compound extracted from the rhizoma of *Curcuma longa* L. which possesses comprehensive bioactivities. The present study aimed to evaluate whether neurobehavioral deficits, neuroendocrine-immune dysregulations and cerebral microcirculation dysfunction, are part of the initial stages of cerebral ischemia in individuals suffering from carotid atherosclerosis resulting from a high cholesterol diet (HCD) and if they could be tested using a comorbid animal model. Furthermore, the utility of this model will be examined following the administration of curcumin. Adult wild-type SD rats were fed a regular diet or HCD and supplemented with either vehicle or curcumin for 4 weeks. Carotid injury was induced by an air-drying endothelial denudation method at the end of the second week. Plasma cholesterol, carotid pathomorphology, neurobehavioral tests, and neuroendocrine-immune parameters were measured. We found higher plasma levels of total cholesterol (TC), triglyceride (TG), low-density lipoprotein-cholesterol (LDL-C), intima and media (I/M) ratio, but lower high-density lipoprotein-cholesterol (HDL-C), spatial learning and memory capacity impairment, elevated NPY expression in the hypothalamus, increased plasma concentration of leptin, upregulated TNF-α, IL-1β, and CRP in the circulation as well as TNF-α and IL-1β in the cerebral cortex, plus enhanced ICAM-1, VCAM-1, and E-selectin in cerebral microvessels in HCD-fed model rats. All these alterations were ameliorated by curcumin. These results suggest that a comorbid rat model was effectively developed by HCD and carotid injury.

## Introduction

Carotid artery atherosclerosis, as one of the major causes of cerebrovascular disease, has been implicated in the etiology of stroke, cognitive impairment, and dementia (Muller et al., 2007; Carrington Rice et al., 2009; Romero et al., 2009). Epidemiological evidence exists to support the idea that neurological dysfunction and carotid artery atherosclerosis will coexist for several years until the first evident neurological symptoms appear (Colín-Castelán and Zaina, 2019). Growing data have demonstrated that hemodynamic changes in the cerebral circulation may be one of the important mechanisms underlying the association between carotid atherosclerosis and brain damage (Muller et al., 2011). Patients with carotid atherosclerosis demonstrate an increasing risk of chronic brain hypoperfusion, which may lead to cerebral small-vessel disease or embolic stroke in regions of the brain with reduced regional cerebral blood flow and impaired clearance of emboli (Manolio et al., 1999; Bokkers et al., 2009; Sojkova et al., 2010). Hypercholesterolemia is considered to be one of the major causes of atherosclerosis (Defesche et al., 2017; Yu et al., 2019). Several studies suggest that the vascular effects caused by a high cholesterol diet (HCD) lead to insufficiencies at the cerebral microcirculation level (Stokes et al., 2002; Ishikawa et al., 2004; Stapleton et al., 2010a; Li et al., 2013).

Inflammatory processes and activation of the neuroendocrine systems are considered to constitute important aspects of the etiology and pathophysiology of vascular disease that eventually may cause ischemic stroke (Tu et al., 2013). Based on evidence from experimental and clinical studies, it has been unequivocally unveiled that excessive intake of dietary fats and cholesterol can evoke a basal systemic low-grade inflammation (Hotamisligil, 2006; Cai, 2009; Cai and Liu, 2012). The mechanisms underlying this chronic low-grade inflammation occur through white adipose tissue which serve as the key site that contributes to increased circulatory proinflammatory cytokines (Odegaard and Chawla, 2013). Peripheral cytokines and leukocytes can infiltrate the brain parenchyma due to diet-induced blood–brain barrier dysfunction and induce cytokine production within the brain (Dantzer et al., 2008; Ávalos et al., 2018). Thus, central inflammatory changes are observed after HCD feeding (Miller and Spencer, 2014). An array of studies have shown that neuroendocrine profiles are changed significantly in hypercholesterolemia induced by HCD. This dyslipidemia may change the hypothalamic hormonal milieu, its interaction with other metabolic hormones, increases the release of adipose tissue-derived hormones such as leptin and other factors that can elicit a chronic inflammatory response that is interrelated with a broad array of metabolic homeostasis processes, to eventually influence the regulation of neuroendocrine function (Kim et al., 2007; Morton et al., 2014; Manfredi-Lozano et al., 2016). Moreover, changes in hypothalamic neuropeptide expression are exhibited in response to HCD with respect to the signals of adipose tissue hormones transmitted to the hypothalamic centers (Morton et al., 2006).

Curcumin [1,7-bis (4-hydroxy-3-methoxyphenyl)-1,6-heptadiene-3,5-dione] is a natural active polyphenolic compound extracted from the rhizoma of the perennial plant *Curcuma longa* L. (turmeric). Several *in vitro* and animal intervention studies have reported that curcumin has antioxidant, anti-inflammatory, hypocholesterolemic, neuroprotective, and hormonal regulatory effects (Maheshwari et al., 2006; Prasad et al., 2014; Arablou and Kolahdouz-Mohammadi, 2018; Zou et al., 2021). A number of clinical trials have focused on the role of curcumin in the prevention and treatment of a series of chronic diseases, such as neurological, metabolic, and cardiovascular diseases (Lopresti et al., 2012; Naksuriya et al., 2014; He et al., 2015).

In our previous study, we developed a complex rat model by combining treatments of HCD and carotid air-drying injury, which is likely to represent the early actual progressive chronic cerebral ischemia caused by carotid atherosclerosis combined with hypercholesterolemia. This pathological animal model was observed to have changes in oxidative stress, as well as inflammation in cerebral microvessels and brain parenchyma (Zhang L. et al., 2014). The current study extends previous work by examining the utility of this model for evaluating the role of curcumin in adverse cerebral microcirculation dysfunction and the neurobehavioral and neuroendocrine-immune consequences of the initial stages of cerebral ischemia in organisms suffering from carotid atherosclerosis related to HCD. Given the multifactorial nature of the preexisting disease before an acute ischemic stroke was developed, treatments that target multiple mechanisms of action are likely to enhance therapy efficacy. Curcumin was selected for this study due to its large array of molecular targets of action. We will attempt to explore how neuroendocrine, immune, and inflammatory dysregulations may be some of the first steps in the pathogenesis of cerebral microcirculation dysfunction and neurological deficits related to carotid atherosclerosis, and how desynchronization of neuroendocrine and immune functions may also exert a negative effect on the evolution of acute cerebral ischemia in this complex rat model. The ultimate purpose of the study is to enhance our understanding of whether this comorbid animal model might effectively mimic the early actual progressive aspects of the stroke-prone state in humans and whether it might provide a more authentic preclinical model for testing novel pharmacological approaches.

## Materials and Methods

### Animals, Diets, and Experimental Design

All of the animal procedures and all of the experimentation protocols included in this study were conducted in strict conformity with ARRIVE (Animal Research: Reporting *In Vivo* Experiments) guidelines and were approved by the Ethics Committee of Jining Medical University (approval number JNMC-2020-DW-JC-012). Every effort was carefully made to minimize animal suffering, and blinding was maintained by experimenters until the entire research was terminated.

Male Sprague–Dawley (SD) rats (160–200 g) from Pengyue Experimental Animal Breeding Institute of Jinan, China, were housed in a temperature- and humidity-controlled room (23-24°C, 50–60% humidity) under a 12-h light/dark cycle and specific pathogen-free (SPF) conditions with free access to water and food. The animals were fed regular chow pellets during the adaptation period of 7 days. Then, rats were randomly assigned to three groups (20 rats each). Rats in the vehicle-treated group and curcumin-treated group were fed HCD and gavaged once daily either with vehicle (1% carboxymethyl cellulose sodium) or 200 mg/kg body weight curcumin for 28 days (4 weeks). Rats in the control (CON) group were fed a regular diet (RD) plus an equal volume of vehicle once daily by gavage for 28 days (4 weeks).

At Day 14 (the end of the second week), carotid endothelial injury was performed by using the air-drying method for all three groups, as described previously (Zhang L. et al., 2014). A 30-gauge hypodermic needle was inserted into the left common carotid artery with a hole near the carotid bifurcation so that sterile medical air flowed through the artery for a total of 4 min at a constant rate of 28 ml/min.

The feeding material in this study was either regular commercially prepared pellet chow administered during the adaptation period or an HCD prepared from a regular diet (72.8%), cholesterol (2%), cholic acid (0.2%), yolk powder (15%), and lard (10%) (Zhang L. et al., 2014). Curcumin was dissolved in 1% carboxymethyl cellulose sodium and was administered by oral gavage once daily at a dose of 200 mg/kg body weight at 9:00 a.m. for 28 days (4 weeks). The selection of the dose of curcumin corresponded to previous reports of curcumin treatment and the preliminary experiment in the current research.

**FIGURE 1:**
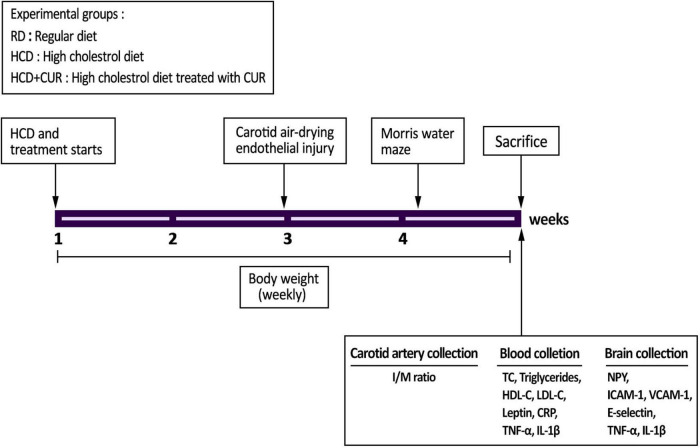
Experimental outline. SD rats were divided into three experimental groups (*n* = 20/each): control group fed a regular diet and vehicle (1% carboxymethyl cellulose sodium, CMC) (RD); group fed a high-cholesterol diet and 1% CMC by gavage (HCD); and group fed a HCD and a daily dose of 200 mg/kg body weight curcumin by gavage (HCD + CUR) for 4 weeks. At the end of the second week, carotid endothelial injury was performed by using the air-drying endothelial denudation method for these three groups. Body weight was monitored throughout the 4-week study period. The Morris water maze was carried out 5 days before sacrifice as indicated in the figure. After sacrifice, biological samples were used for assessments of biochemical, histological, neuroendocrine, and immune-related markers.

A brief summary of the experimental protocol is shown in Figure 1. Two independent experiments were carried out to ensure the reproducibility of the data. Body weight was determined once a week throughout the study. The Morris water maze was carried out five days before sacrifice (*n* = 10 per group). At the end of the study, animals were anesthetized (chloral hydrate 0.04 g/kg, i.p.) and sacrificed by cervical dislocation after overnight fasting and weighing. Blood samples were collected in heparin-coated tubes and centrifuged at 800 g for 10 min, and the plasma was stored at –80°C for inflammation, hormonal, and metabolic parameter analysis (*n* = 10 per group). The hypothalamus (*n* = 4 per group) and cortex tissues (*n* = 10 per group) were immediately frozen in liquid nitrogen until analysis. The injured carotid artery samples were preserved in 4% buffered neutral paraformaldehyde for morphometric analysis (*n* = 6 per group). All of these tests were performed as described in the following sections.

### Morris Water Maze Task

The water maze test was used here as described previously, with some modifications (Ya et al., 2013). Briefly, before beginning acquisition training, rats were allowed a 2-min swim to reduce stress in the pool without platform present. Then, rats were given daily sessions of four acquisition trials (120 s each) over the following four consecutive days with the platform (diameter 10 cm) 1.5 cm beneath the water surface located in the center of a quadrant of the pool for all rats throughout the experiments, and the average of the four acquisition trials was used for data analysis. Rats were released from four different starting positions in a random order facing the edge of the pool to avoid side bias between rats. The escape latency (s) from the releasing point to reach the submerged platform, swimming distance (cm) to the platform, and swimming speed (cm/s) were collected.

One hour after the final acquisition trial (Day 4), mice were given a probe trial of 60 s by removing the platform from the pool. The swimming time spent and distance traveled in the quadrant (where the platform was located previously) were measured. The ratio of time and distance in the target quadrant compared to those in the whole pool were calculated.

### Biochemical Analyses

Metabolic parameters were quantified in rat blood plasma using biochemical kits (Jiancheng Company, Nanjing, China) for total cholesterol (TC), low-density lipoprotein cholesterol (LDL-C), high-density lipoprotein cholesterol (HDL-C), and triglycerides (TGs) according to the manufacturer’s instructions. Plasma leptin as an endocrine parameter was determined by a Rat Leptin ELISA Kit (Boster Biological Technology Co., Ltd., Wuhan, China) with a sensitivity threshold of 0.3 ng/mL. Inflammation status was assessed in plasma and supernatants of brain homogenates by ELISA detection kits [comprising tumor necrosis factor-alpha (TNF-α), interleukin-1β (IL-1β), and C-reactive protein (CRP): Boster Biological Technology Co., Ltd., Wuhan, China] according to the manufacturer’s instructions.

### Carotid Pathomorphological Detection

Six rats from each group were sacrificed 14 days after surgery for morphometric analysis. Four cross sections (5 mm thick) from the central part of excised segments of the injured carotid arteries in each rat were used, and all samples were stained with hematoxylin and eosin (HE). The quantification of intimal thickening was assessed by Image Pro^®^ Plus 5.0 software (Media Cybernetics, Inc., Bethesda, MD, United States), and the extent of carotid intimal lesions was calculated as a ratio of the intima and media (I/M).

### Cerebral Microvessel Isolation

Cerebral microvessels were isolated from rat brains as previously described (Zhang L. et al., 2014). In brief, the left brain cortex was dissected, cleared of pia mater, homogenized by hand in a Dounce tissue grinder in 3 vol ice-cold PBS (0.01 mol/L, pH 7.4), and then centrifuged at 1,000 g for 10 min at 4°C. The supernatant of brain homogenates was stored at –80°C for further assays of inflammation status. The final pellet was filtered with a strong stream of cold PBS through a nylon mesh screen (50 mm). The microvessel fraction was removed from the screen, resuspended in PBS, and diluted to 2 mg protein/ml. Protein content was determined utilizing Lowry’s method (Lowry et al., 1951), and the homogenates were used for the assays of intercellular adhesion molecule 1 (ICAM-1), vascular cell adhesion molecule 1 (VCAM-1), and E-selectin. Aliquots were separated for microvessel morphologic evaluations on dried smears fixed with 10% formaldehyde and stained with toluidine blue.

### Western Blot Analysis

Total protein samples extracted from the dissected hypothalamic tissue and the microvessel fraction were subjected to electrophoresis, transferred onto PVDF membranes, and incubated with primary antibodies comprising: mouse anti-neuropeptide Y (NPY) monoclonal antibodies (Santa Cruz Biotechnology, Inc., United States), rabbit anti-VCAM-1 polyclonal antibody (Cell Signaling Technology, United States), rabbit anti-ICAM-1 polyclonal antibody (Cell Signaling Technology, United States), and mouse anti-E-selectin monoclonal antibody (Santa Cruz Biotechnology, Inc., United States). β-Actin antibody was used for sample normalization to express the final result as the intensity ratio (% sham-operated control).

### Statistical Analysis

All data were analyzed using SPSS statistical software, and results were expressed as mean ± standard error (SE). Data for escape latency, swimming distance, and swimming speed in Morris water maze test were analyzed using a two-way repeated analysis of variance (ANOVA), with treatment as between factors and day as within factors, and treatment effect was performed using a *post hoc* Duncan’s test. One-way ANOVA with subsequent Duncan’s test as *post hoc* analysis was used for statistical analysis of all the other data. A *P*-value < 0.05 was considered to indicate statistical significance.

## Results

### Body Weight and Plasma Lipid Profiles

**FIGURE 2:**
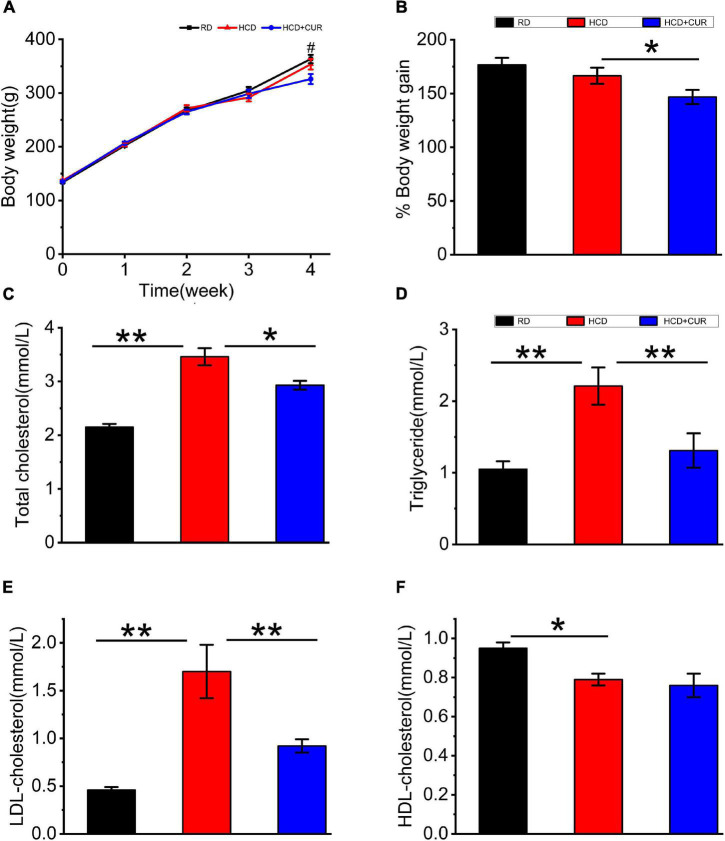
Changes in body weight and plasma lipid profiles in rats. **(A)** Weekly body weight (g); **(B)** body weight gain (%); **(C)** total cholesterol (TC); **(D)** triglyceride (TG); **(E)** LDL-cholesterol (LDL-C); **(F)** HDL-cholesterol (HDL-C). RD, control group; HCD, high cholesterol diet group; HCD + CUR, high cholesterol diet receiving a daily dose of 200 mg/kg body weight curcumin by gavage for 4 weeks. Carotid injury was induced by an air-drying endothelial denudation method at the end of the second week. Data are representative of two independent experiments and expressed as mean ± SE, *n* = 10 for each group. ^#^*P* < 0.05, vs. HCD group; *P < 0.05, **P < 0.01.

Rats were placed on HCD or RD for 4 weeks, and their weights were measured once a week. There was a gradual increase in the body weight of rats in all the groups over 4 weeks on their respective diets (Figure 2A). The body weights did not differ between the RD-fed control and HCD-fed model groups, as body weight increased by 176.7% in RD-fed rats and 166.5% in HCD-fed rats, which corresponds to an absolute increase of 229.7 g in RD-fed rats and 216.5 g in HCD-fed rats. The administration of CUR attenuated the increase in body weight gain of HCD-fed rats by approximately 12%, showing a relative weight gain of 146.77%, which corresponds to 190.8 g at the end of the intervention (*p* < 0.05) (Figure 2B).

Plasma TC, TG, LDL-C, and HDL-C (Figures 2C–F) in RD-fed rats were 2.15 ± 0.06, 1.05 ± 0.11, 0.46 ± 0.03, and 0.95 ± 0.03 mmol/l, respectively. Plasma lipid profiling data showed that following 4 weeks on HCD, rats in the HCD-fed model group showed a significant increase in plasma levels of TC, TG, and LDL-C (*p* < 0.01) and a significant decrease in plasma levels of HDL-C (*p* < 0.05). Treatment with CUR led to a significant reduction in the levels of plasma TC, TG, and LDL-C in HCD-fed rats by 15% (*p* < 0.05), 44% (*p* < 0.01), and 46% (*p* < 0.01), respectively. However, the level of plasma HDL-C did not show a significant difference in response to CUR.

### The Extent of Rat Carotid Intimal Thickening

**FIGURE 3:**
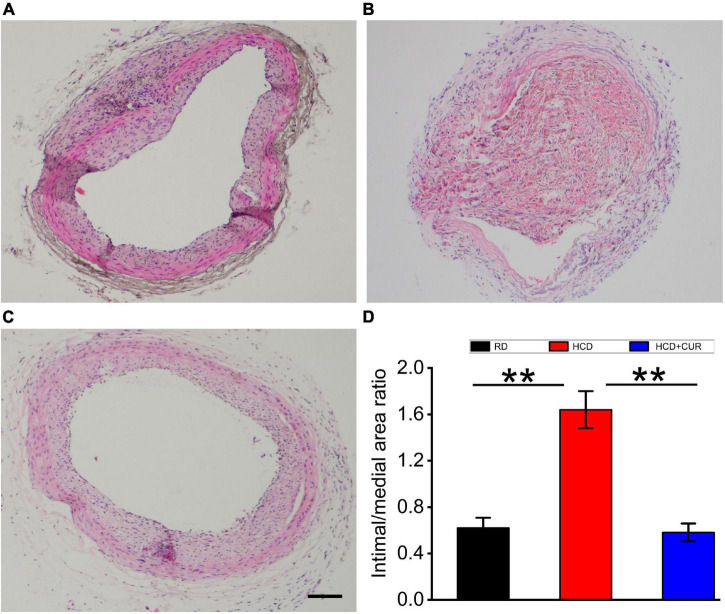
Changes in pathomorphology of common carotid artery in rats. H&E staining of representative cross-sections at the center of the injured region of carotid arteries, **(A)** RD group; **(B)** HCD group; **(C)** HCD + CUR group; **(D)** image analysis of carotid stenosis ratios (%) expressed as intima/media area × 100% (I/M ratio). RD, control group; HCD, high cholesterol diet group; HCD + CUR, high cholesterol diet receiving a daily dose of 200 mg/kg body weight curcumin by gavage for 4 weeks. Carotid injury was induced by an air-drying endothelial denudation method at the end of the second week. Data are representative of two independent experiments and are expressed as mean ± SE, *n* = 6 for each group. **P < 0.01. Scale bar in **(A–C)** = 100 μm.

The results of neointimal hyperplasia that causes cumulative carotid lumen stenosis were quantified by pathomorphometric analysis of carotid artery cross sections in the rats at Day 14 after carotid endothelial injury (Figure 3). In the HCD-fed model rats, the injured carotid intimal thickening was more extensive than that in the RD-fed control rats (*p* < 0.01). The carotid stenosis ratio in HCD-fed model rats was increased by 66% compared to that in the RD-fed control group. The increase in intimal thickness was significantly reduced by oral administration of CUR (*p* < 0.01). Compared to that in the HCD-fed model group, the I/M ratio (1.64 ± 0.16 in the HCD-fed model group vs. 0.58 ± 0.08 in the CUR group, *P* < 0.05) was significantly decreased in the CUR treatment group.

### Spatial Learning and Memory in Morris Water Maze Test

**FIGURE 4:**
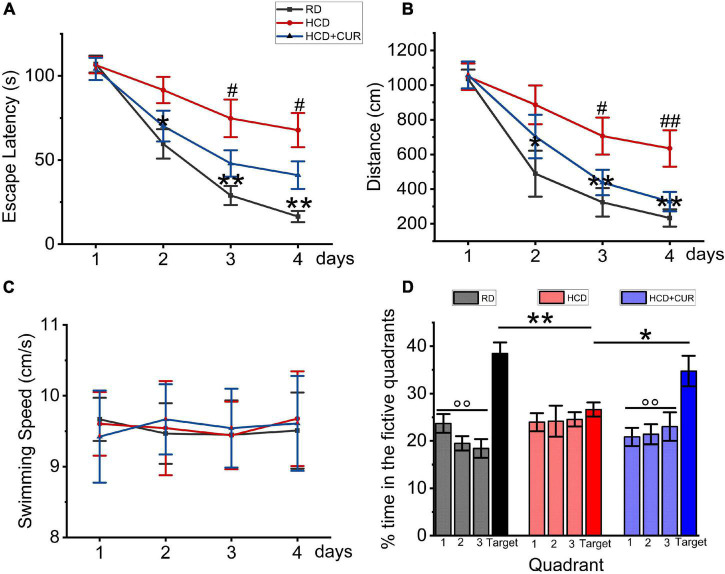
Changes in spatial learning and memory in Morris water maze test in rats. RD, control group; HCD, high cholesterol diet group; HCD + CUR, high cholesterol diet receiving a daily dose of 200 mg/kg body weight curcumin by gavage for 4 weeks. Carotid injury was induced by an air-drying endothelial denudation method at the end of the second week. **(A)** Comparison of mean escape latencies to platform during four training days; data are expressed as mean ± SE, *n* = 10 for each group. *P < 0.05, **P < 0.01, vs. control group. ^#^*P* < 0.05, vs. HCD group. **(B)** Comparison of mean swimming distances during four training days; Data are expressed as mean ± SE, *n* = 10 for each group. *P < 0.05, **P < 0.01, vs. control group. ^#^*P* < 0.05, ^##^*P* < 0.01, vs. HCD group. **(C)** Comparison of swimming speed during 4 training days; Data are expressed as mean ± SE, *n* = 10 for each group. **(D)** Comparison of the percent of searching time spent in the quadrant where the platform was removed for probe trial. Data are representative of two independent experiments and are expressed as mean ± SE, *n* = 10 for each group. *P < 0.05; **P < 0.01.°°*P* < 0.01, vs. target quadrant.

The Morris water maze test was conducted to establish whether spatial learning and memory capacity impairment develop due to progressive chronic cerebral ischemia caused by HCD and carotid air-drying injury in this complex rat model, as well as the effects of CUR on learning and memory. The results of the acquisition experiments from Day 1 to Day 4 are shown in Figures 4A–C. In these experiments, data were analyzed over days by beginning with the average of the four acquisition trials within days. Rats learned the task progressively, as indicated by decreasing swimming latencies (Figure 4A), *F*(3,27) = 39.833, *p* < 0.001, and distances to the platform (Figure 4B), *F*(3,27) = 51.747, *p* < 0.001. This was confirmed by two-factor ANOVA of the escape latency and swimming distance data, indicating the main effect of day. Differences were also detected between groups, *F*(2,18) = 11.902, *p* = 0.001 and *F*(2,18) = 6.407, *p* = 0.008, respectively, but no group × day interaction. *Post hoc* analysis of the simple main effect of group revealed significant differences between RD-fed control, HCD-fed model, and CUR-treated group for the latency and distance measure. Latencies and swimming distances of HCD-fed model rats were significantly higher than those of RD-fed control rats on Days 2–4 (respectively, *P* = 0.014, *P* = 0.001, *p* < 0.001 and *P* = 0.031, *P* = 0.005, *P* = 0.001), suggesting that model rats took an extremely long time to find the hidden platform. No significant difference was noted concerning swim speed (18.7 ± 1.7 vs. 19.7 ± 1.5 cm/s) (Figure 3C), suggesting that the locomotor skills did not differ among groups. Thus, HCD-fed model rats exhibited impaired learning ability in the MWM task. However, CUR treatment led to significantly shortened escape latency and swimming distance compared to the HCD-fed model group. Figure 4D depicts the results of the probe test in which the platform was removed from the target quadrant of the tank. ANOVA revealed that there was no preference for the target quadrant relative to chance level [*t*(18) = 0.529; *P* > 0.5] by the HCD-fed model group (*F* < 0.5; *P* > 0.5), which may be affected by the low memory consolidation shown in the acquisition experiments or may be indicative of memory extinction. *Post hoc* analyses confirmed that the HCD-fed model rats spent a significantly lower percentage of time searching in the target area than RD-fed control animals (*P* < 0.0001). However, the CUR treatment group with a quadrant effect [*F*(3,28) = 5.34, *P* = 0.005] displayed a significantly higher percentage of time in the target quadrant than HCD-fed model rats (*P* = 0.045, *P* = 0.001, and *P* = 0.017). Taken together, the neurobehavioral data suggested that, relative to RD-fed control animals, HCD-fed model rats showed a lower encoding of spatial information and did significantly worse during the retention test.

### Expression Levels of Specific Endothelial Adhesion Molecules in the Cerebral Microvasculature

**FIGURE 5:**
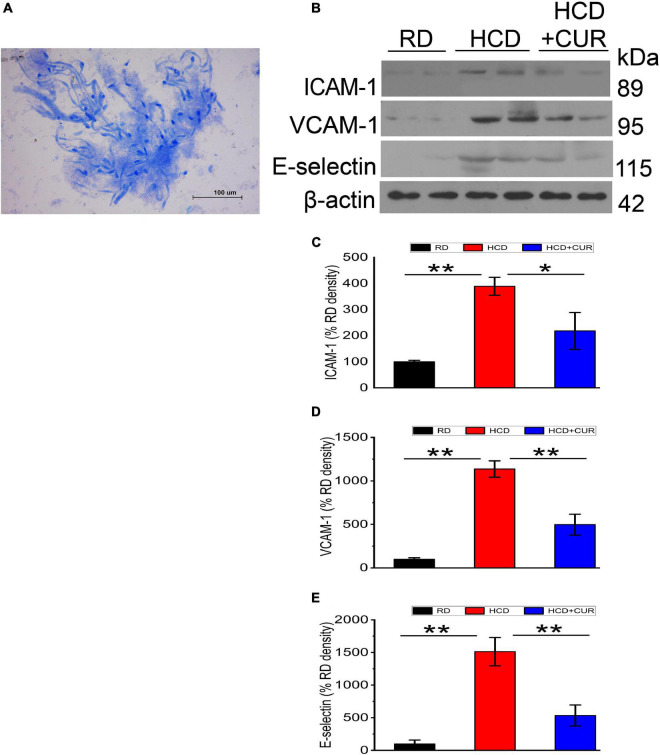
Changes in expression levels of ICAM-1, VCAM-1, and E-selectin in the cerebral microvasculature in rats. **(A)** Light photomicrograph of the isolated microvascular fraction from rat cerebral cortex. Scale bar = 100 μm. **(B–E)** Representative western blot of ICAM-1, VCAM-1, and E-selectin in cerebral microvessels. Quantification of ICAM-1, VCAM-1, and E-selectin normalized with β-actin. RD, control group; HCD, high cholesterol diet group; HCD + CUR, high cholesterol diet receiving a daily dose of 200 mg/kg body weight curcumin by gavage for 4 weeks. Carotid injury was induced by an air-drying endothelial denudation method at the end of the second week. Data are representative of two independent experiments and are expressed as mean ± SE, *n* = 4 for each group. *P < 0.05, **P < 0.01.

Endothelial cell activation in the cerebral microvasculature in response to HCD and an altered brain flow environment caused by carotid atherosclerosis has been demonstrated previously (Nakashima et al., 1998; Ishikawa et al., 2004; Yin et al., 2015). In the present study, microvessels were isolated from the brains of rats, and the protein expression levels of specific endothelial adhesion molecules, including ICAM-1, VCAM-1, and E-selectin, were evaluated by western blotting. The results (Figure 5) showed that ICAM-1, VCAM-1, and E-selectin in cerebral microvessels of HCD-fed model rats were upregulated by 3. 9–, 11. 4–, and 15.1-fold, respectively, compared with the RD-fed control group (*P* < 0.01), suggesting that changes in plasma lipid levels and cerebral circulation hemodynamics appeared to regulate these three adhesion molecules in the cerebral microvasculature and endothelial cells were activated in the cerebral microvessels in this complex rat model of experimental ischemic stroke. The enhancement of ICAM-1, VCAM-1, and E-selectin was inhibited by 44, 56.2, and 64.7%, respectively, by CUR treatment.

### Determination of NPY Level in the Hypothalamus and Plasma Leptin and Proinflammatory Markers in the Circulation and Cerebral Cortex

**FIGURE 6:**
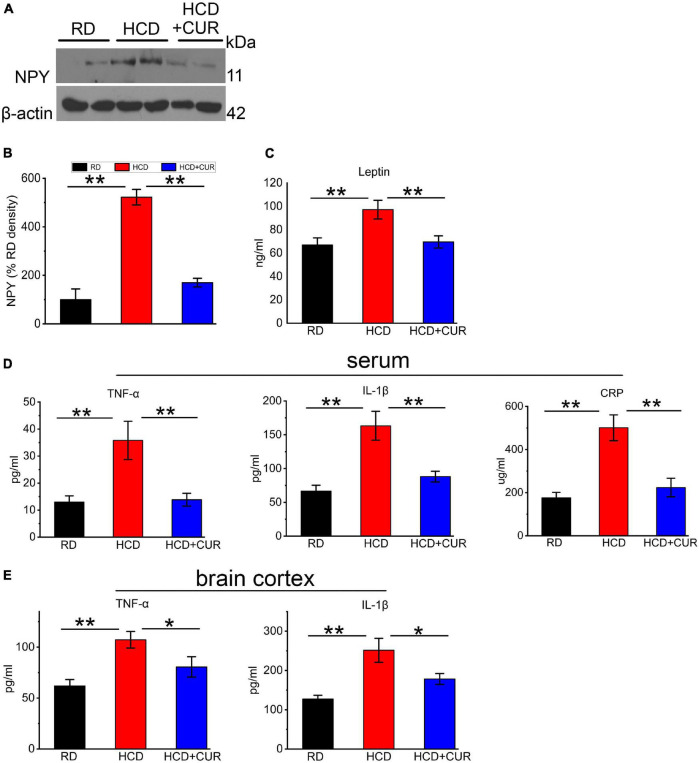
Changes in NPY level in hypothalamus and leptin, TNF-α, IL-1β, and CRP in plasma and/or cerebral cortex in rats. **(A,B)** Representative western blot of NPY in hypothalamus. Quantification of nuclear NPY normalized with β-actin. Data are expressed as mean ± SE, *n* = 4 for each group. **(C)** Leptin in plasma; **(D)** TNF-α, IL-1β, and CRP in plasma; **(E)** TNF-α and IL-1β in cerebral cortex. RD, control group; HCD, high cholesterol diet group; HCD + CUR, high cholesterol diet receiving a daily dose of 200 mg/kg body weight curcumin by gavage for 4 weeks. Carotid injury was induced by an air-drying endothelial denudation method at the end of the second week. Data are representative of two independent experiments and are expressed as mean ± SE, *n* = 10 for each group. *P < 0.05, **P < 0.01.

Neuroendocrine and inflammatory changes underlie comorbidity factors such as carotid atherosclerosis and hypercholesterolemia, which may lead to enhanced cerebral tissue injury, and have potential consequences for the pathophysiology of cerebrovascular diseases, especially stroke caused by arterial occlusion or ischemia. We therefore analyzed NPY levels in the hypothalamus, as well as plasma leptin and proinflammatory cytokines in the circulation and cerebral cortex. To investigate whether HCD and an altered brain flow environment had general effects on NPY expression in the hypothalamus of the complex rat model of experimental ischemic stroke, we checked NPY expression in the hypothalamus using western blot analysis. As seen in Figures 6A,B, NPY expression was significantly increased 5.2-fold in the HCD-fed model rats compared with the RD-fed control rats (*P* < 0.01), which was inhibited by CUR treatment (*P* < 0.01). HCD-fed model rats showed significantly higher plasma concentrations of leptin (*p* < 0.01) (Figure 6C) than RD-fed control rats. The administration of CUR led to a significant reduction (*p* < 0.01) in plasma leptin in HCD-fed model rats. We analyzed proinflammatory marker levels in our rats and determined that the proinflammatory markers were elevated in the circulation as well as in the cerebral cortex of HCD-fed model rats, suggesting that HCD and carotid atherosclerosis led to increased peripheral and cerebral immune responses, which went along with neuroendocrine changes in this complex rat model. HCD-fed model rats exhibited 63.7% elevated plasma TNF-α, 59.5% elevated plasma IL-1β, and 185.0% elevated plasma CRP levels compared to the RD-fed control rats (*P* < 0.01). Treatment with CUR decreased the levels of TNF-α, IL-1β and CRP by 61.3, 46, and 55.5%, respectively (Figure 6D). TNF-α levels were 42.3% higher in brain cortex lysates of HCD-fed model rats, at the protein level, than in RD-fed control rats, while the level of IL-1β increased by 29.1%. In the brain cortex lysates, a similar profile was observed by CUR treatment, in which the levels of TNF-α and IL-1β were decreased by 24.9 and 29.1%, respectively (Figure 6E).

## Discussion

Growing evidence shows that hypercholesterolemia and carotid artery disease are involved in the high-risk factor profile of cerebrovascular disease (Röther et al., 2008). Many key physiological systems, such as the nervous, endocrine, and immune systems, are associated with progressive changes in pathological states, such as the development and/or exacerbation of cerebrovascular disease, in which the imbalance of the neuroendocrine-immune relationship, as well as cognitive impairment, appears in setting up the progression of the physiopathological scenario. Our previous work developed a new complex rat model that better resembled early actual progressive chronic cerebral ischemia. This complex rat model showed abnormal blood lipid profiles, atherosclerosis in the carotid artery, oxidative stress, and inflammatory changes in cerebral microvessels and brain parenchyma. These characteristics were more pronounced than those observed in either the hypercholesterolemia alone or the carotid injury group, mirroring more natural conditions of the pathophysiology before an acute ischemic insult was developed (Zhang L. et al., 2014). Along the line, we asked whether in this complex rat model of experimental ischemic stroke induced by the method of air-drying injury of the left common carotid artery based on 4 weeks of HCD feeding, neurobehavioral alterations, systemic immune response stimulation, abnormal neuroendocrine metabolism, such as neuroinflammation, metabolism, and hemodynamics related cerebral microvascular endothelial activation, could be involved in the early stages of stroke in the animals already exposed to carotid atherosclerosis and concomitant dietary hypercholesterolemia. In the present study, we indicate several novel findings: (1) HCD-fed model rats showed declined acquisition of a learning and memory paradigm in the MWM, indicating that cognitive dysfunctions in rats could be induced by chronic cerebral hypoperfusion as well as hypercholesterolemia; (2) we identified that ICAM-1, VCAM-1, and E-selectin in cerebral microvessels of HCD-fed model rats were upregulated, suggesting that the combination of sustained hypoperfusion and hypercholesterolemia promoted cerebral microvascular endothelial activation; and (3) carotid atherosclerosis and concomitant dietary hypercholesterolemia induced alterations in the expression of NPY in hypothalamus, the level of plasma leptin and proinflammatory markers such as TNF-α, IL-1β, and CRP in the circulation, as well as TNF-α and IL-1β in the cerebral cortex. These changes suggest perturbations in the bidirectional communication between neuronal, endocrine, and immune systems correlated with cerebral ischemia-associated comorbidity factors such as carotid atherosclerosis and hypercholesterolemia that might be related to high risk for future cerebrovascular events (Colín-Castelán and Zaina, 2019); interventions with curcumin helped to beneficially attenuate carotid lumen stenosis, modulate lipid metabolism, improve learning and memory impairments, ameliorate inflammation reactions in the cerebral microvasculature, and regulate neuroendocrine-immune changes in this complex rat model of experimental ischemic stroke.

It has been well established that hypercholesterolemia is a substantial risk factor associated with atherosclerosis, as elevated cholesterol has been shown to interrupt and modify vascular structure and function (Stapleton et al., 2010a,b). Accordingly, we observed the most prominent changes in the combined model with regard to the extent of carotid atherosclerosis and stenosis, which are believed to be one of the mechanisms inducing or contributing to hemodynamic compromise in the cerebral cortex microcirculation (Bokkers et al., 2009), indicating that hypercholesterolemia promoted atherosclerotic plaque development, which was well correlated with previous reports (Chung et al., 2003). The role of hypoperfusion caused by narrowing of the carotid lumen has a potent impact on the upregulation of inflammatory-related pathological damage in cerebral microvasculation. The inflammatory response is initiated by cytokine activation (TNF-α and IL-1β). These cytokines mediate the development of acute phase reactants, such as CRP, and the release of cell adhesion molecules, causing microvascular occlusion (Rodríguez-Yáñez and Castillo, 2008). The elevated plasma level of acute phase reactant C-reactive protein (CRP) has been reported to be associated with a higher risk of stroke (Rost et al., 2001). Inflammatory cytokines (TNF-α and IL-1β) and adhesion cell molecules are useful to evaluate the risk of stroke and have fundamental roles in the pathophysiology of brain ischemia (Blum et al., 2006; Wang et al., 2006; Chen et al., 2014). Endothelial dysfunction reflected by elevated release of proinflammatory factors and activation of cell adhesion molecules in turn causes reduced regional cerebral blood flow, which enhances hypoperfusion, resulting in a vicious cycle (Daulatzai, 2017). As such, experimental microvascular studies have clearly provided strong support for the notion that two molecular events that enhanced cell adhesion-dependent signaling and/or increased secretion of proinflammatory cytokines are involved in the persistent activation of endothelial cells of cerebral microcirculation as they respond to hypercholesterolemia (Stokes et al., 2002; Ishikawa et al., 2004; Yang et al., 2008). We analyzed adhesion molecule expression in the cerebral microcirculation in this combined model. Taking into account the progressive reduction in cerebral blood flow caused by carotid atherosclerosis and stenosis that was aggravated by a high cholesterol chow, our data suggested that the sustained hypoperfusion and hypercholesterolemia-induced changes in the cerebral microvasculature were synergized, resulting in cerebral microvascular endothelial activation manifested by upregulation of cell surface adhesion molecules, including ICAM-1, VCAM-1, and E-selectin. In the present study, we reported that lipid metabolism and carotid lumen stenosis were ameliorated by CUR administration in parallel with reductions in cell inflammation, it has been shown that hypercholesterolemia in conjunction with chronic hypoperfusion disrupts blood brain barrier integrity, and endothelial activation enables the infiltration of peripheral immune cells to sites of ischemic brain damage (Dirnagl et al., 1999; Ayata et al., 2013; Lynch et al., 2013). Several studies have found a link between systemic inflammation and central inflammation, as peripheral cytokines can have an action on the brain to induce the local expression of cytokines (Dantzer et al., 2008; Byeong Tak et al., 2012; Boitard et al., 2014). Our data showed central inflammation as reflected by the amounts of TNF-α and IL-1β with the most prominent increase in brain parenchyma in the combined setting, and similar regulations of TNF-α, IL-1β, and CRP expression have been observed in plasma in carotid injured rats on HCD, as previously suggested that this inflammatory microenvironment might be partially induced by hypercholesterolemia (Drake et al., 2011). We did not observe these regulations in the RD-fed control group, as cell adhesion molecule expression in cerebral microcirculation, as well as proinflammatory markers in brain parenchyma and in plasma were near basal expression levels, suggesting that hypercholesterolemia decreased the threshold for induction of central and peripheral immune activation. In addition, these classical proinflammatory cytokines, such as TNF-α and IL-1β, may be involved in learning and memory (Spyridaki et al., 2014; Rizzo et al., 2018). CUR also seemed to play an important role in reducing TNF-α and IL-1β expression in the brain parenchyma, plus TNF-α, IL-1β, and CRP in plasma.

It is common and etiologically crucial for comorbidities such as carotid atherosclerosis and hypercholesterolemia in stroke patients, to induce a “primed” inflammatory environment in advance of a cerebrovascular event in the brain, as previously reported (Drake et al., 2011; Purkayastha and Cai, 2013). Taking into account the omnipresent nature of comorbidities preceding stroke, such as inflammation, there have been few studies involving these in experimental stroke research (Terao et al., 2008; Li et al., 2013). Furthermore, few preclinical studies emphasize the role of the neuroendocrine-immune system in the complex animal model of carotid atherosclerosis and hypercholesterolemia. Overnutrition, such as excessive intake of dietary cholesterol, has been implicated as one of the prominent factors associated with metabolic alterations. In concurrence with metabolic dysfunctions, overfeeding has been well accepted to trigger an inflammatory response, and the release of inflammatory factors such as TNF-α, IL-1β, CRP, and soluble adhesion molecules have also been causally linked to metabolic disorders (Hotamisligil et al., 1993; Herbert Haught et al., 1996; Koh et al., 2005; Ingelsson et al., 2008). The hypothalamus, which drives the central regulation of metabolic homeostasis, exerts its actions via metabolic cue sensing, as well as neurohormonal and neurotransmitter system modulation to regulate energy balance. In the hypothalamus, NPY has been recognized to be involved in the regulation of many physiological functions, such as food intake, memory processing, and cognition (Decressac and Barker, 2012; Beck and Pourié, 2013; Zhang W. et al., 2014). The neuroregulatory mechanisms associated with NPY are mediated by the molecular and cellular integration of endocrine signals originating from peripheral tissues. Leptin, as a signaling molecule, is produced by adipose tissue, released into circulation and transported across the BBB to the brain, where it contributes to energy homeostasis by decreasing food intake and increasing energy expenditure (JéQuier, 2002; Coll et al., 2007). We showed higher amounts of leptin in the circulation in HCD-fed model rats, suggesting leptin insensitivity or resistance (Myers et al., 2008) in this complex model, which were well documented in previous reports that most overweight people and diet-induced common obesity rodent models had enhanced levels of circulating leptin (Spiegelman and Flier, 2001; Enriori et al., 2007). Moreover, increased NPY levels in the hypothalamus were found in HCD-fed model rats. CUR normalized alterations in leptin levels, and the same trend was observed for NPY expression. These results indicated that this complex model exhibited neuroendocrine dysfunction depending on the disordered alteration of the leptin/hypothalamic circuits by integrating neural and endocrine factors.

We demonstrated dysregulation of neuroendocrine factors (NPY and leptin), proinflammatory markers (TNF-α, IL-1β, and CRP), and endothelial adhesion molecules (ICAM-1, VCAM-1, and E-selectin) in this comorbid rat model harboring metabolic and/or systemic vascular disease, plus that parallel variations may be present in patients with clinical risk factors for cerebrovascular disease. In addition, the present study indicates that CUR beneficially regulates the risk factor profile associated with cerebrovascular disease and retards the progression of chronic cerebral ischemia caused by carotid atherosclerosis combined with hypercholesterolemia. The mechanism is mainly that the administration of CUR modulates lipid metabolism and attenuates carotid lumen stenosis, presumably influencing neuroendocrine-immune changes, thereby attenuating cognitive dysfunction.

## Conclusion

In conclusion, we have demonstrated the role of risk factors for stroke, which involve hypercholesterolemia and carotid artery atherosclerosis, in the regulation of neuroendocrine-immune changes and the associated neurobehavioral phenotype, such as in cognitive dysfunction. Our translational approach has built a comorbid rat model that mimics the mechanism of the human stroke-prone state, and this appropriate rat model could help provide insight for future preclinical studies of novel therapeutic developments in the area of comorbidities proceeding stroke.

## Data Availability Statement

The raw data supporting the conclusions of this article will be made available by the authors, without undue reservation.

## Ethics Statement

The animal study was reviewed and approved by the Ethics Committee of Jining Medical University (approval number JNMC-2020-DW-JC-012).

## Author Contributions

BY: supervision and performance of experiments, data analysis, and manuscript preparation. XL: statistical analysis and manuscript preparation. JW, TY, HW, QX, QW, XM, and XD: performance of experiments. MZ: performance of experiments and manuscript preparation. YG and HZ: supervision and performance of experiments. HX: supervision of the study and proofreading of the manuscript. All authors contributed to the article and approved the submitted version.

## Conflict of Interest

The authors declare that the research was conducted in the absence of any commercial or financial relationships that could be construed as a potential conflict of interest.

## Publisher’s Note

All claims expressed in this article are solely those of the authors and do not necessarily represent those of their affiliated organizations, or those of the publisher, the editors and the reviewers. Any product that may be evaluated in this article, or claim that may be made by its manufacturer, is not guaranteed or endorsed by the publisher.
